# Establishment of an indicator framework for global One Health Intrinsic Drivers index based on the grounded theory and fuzzy analytical hierarchy-entropy weight method

**DOI:** 10.1186/s40249-022-01042-3

**Published:** 2022-12-08

**Authors:** Jiaxin Feng, Zhaoyu Guo, Lin Ai, Jingshu Liu, Xiaoxi Zhang, Chunli Cao, Jing Xu, Shang Xia, Xiao-Nong Zhou, Jin Chen, Shizhu Li

**Affiliations:** 1 National Institute of Parasitic Diseases, Chinese Center for Disease Control and Prevention (Chinese Center for Tropical Diseases Research), NHC Key Laboratory of Parasite and Vector Biology, WHO Collaborating Centre for Tropical Diseases, National Center for International Research on Tropical Diseases, Shanghai, 200025 China; 2 School of Global Health, Chinese Center for Tropical Diseases Research, Shanghai Jiao Tong University School of Medicine, Shanghai, 200025 China

**Keywords:** Global One Health, Intrinsic drivers index, Indicator, Grounded theory, Fuzzy analytical hierarchy process, Entropy weight method

## Abstract

**Background:**

One Health has become a global consensus to deal with complex health problems. However, the progress of One Health implementation in many countries is still relatively slow, and there is a lack of systematic evaluation index. The purpose of this study was to establish an indicator framework for global One Health Intrinsic Drivers index (GOH-IDI) to evaluate human, animal and environmental health development process globally.

**Method:**

First, 82 studies were deeply analyzed by a grounded theory (GT) method, including open coding, axial coding, and selective coding, to establish a three-level indicator framework, which was composed of three selective codes, 19 axial codes, and 79 open codes. Then, through semi-structured interviews with 28 health-related experts, the indicators were further integrated and simplified according to the inclusion criteria of the indicators. Finally, the fuzzy analytical hierarchy process combined with the entropy weight method was used to assign weights to the indicators, thus, forming the evaluation indicator framework of human, animal and environmental health development process.

**Results:**

An indicator framework for GOH-IDI was formed consisting of three selective codes, 15 axial codes and 61 open codes. There were six axial codes for “Human Health”, of which “Infectious Diseases” had the highest weight (19.76%) and “Injuries and Violence” had the lowest weight (11.72%). There were four axial codes for “Animal Health”, of which “Animal Epidemic Disease” had the highest weight (39.28%) and “Animal Nutritional Status” had the lowest weight (11.59%). Five axial codes were set under “Environmental Health”, among which, “Air Quality and Climate Change” had the highest weight (22.63%) and “Hazardous Chemicals” had the lowest weight (17.82%).

**Conclusions:**

An indicator framework for GOH-IDI was established in this study. The framework were universal, balanced, and scientific, which hopefully to be a tool for evaluation of the joint development of human, animal and environmental health in different regions globally.

**Supplementary Information:**

The online version contains supplementary material available at 10.1186/s40249-022-01042-3.

## Background

With the deepening of global integration, under the influence of factors such as increased population mobility and intensified environmental change, global public health emergencies become more frequent [1–3]. Human health is closely linked to animals and the ecological environment [4–6], for instance, 60% of known human infectious diseases are zoonotic [7–10], and about 70% of new zoonotic diseases originate in wild animals [11]. Thus, some scholars put forward the concept of One Health, which integrates human, animal, and environmental health to carry out health promotion work [12, 13], the American Veterinary Medical Association first established the One Health Action group in 2007. Based on One Health concept, in 2010, the World Health Organization (WHO), the World Organization for Animal Health (OIE), and the Food and Agriculture Organization of the United Nations (FAO) co-signed an agreement to “sharing responsibilities and coordinating global activities to address health risks at the animal-human-ecosystems interface” [14]. In 2021, the One Health High Level Expert Panel (OHHLEP), organised by experts from  the FAO, OIE, WHO, and the United Nations Environment Programme (UNEP)’s One Health High Level Expert Panel (OHHLEP) officially defined One Health as “an integrated, unifying approach that aims to sustainably balance and optimize the health of people, animals and ecosystems” [15].

Studies have clarified that global One Health index (GOHI) should consist of external drivers index, intrinsic drivers index and core drivers index, to regard the coordinated development of human, animal and environmental health as the target interface, the coordinated development of One Health practice process need external drivers factors such as society, economy, culture, also need core drivers factors such as zoonotic disease prevention and control process, food chain and food safety, prevention and control of microbial resistance and adaptation to climate change [16, 17]. However, there is no unified evaluation standard for One Health Intrinsic Drivers factors in all countries around the world, especially, One Health governance, including human, animal and environmental health development process, is not sufficient in developing countries [18, 19]. At present, there is little studies on health-related evaluation systems around the world, the sustainable development report (SDR) released by the United Nations, the Institute for Health Metrics and Evaluation the global burden of diseases database (IHME-GBD) established by Washington University, and the environmental performance index (EPI) proposed by Yale University study health-related evaluation systems from the aspects of economic development, disease burden, and ecological environment. However, those studies are limited to specific scientific fields of One Health. Recently, the GOHI, a potential assessment tool for One Health performance, was released by the expert group form Shanghai Jiaotong Univercity firstly in the world [16, 20]. As one of important component, the framework for evaluation of global One Health Intrinsic Drivers index (GOH-IDI) has not been reported. Therefore, construction such a framework for GOH-IDI deserves a more detailed study.

Grounded theory (GT) is a qualitative research method proposed by the American scholars, Anselm Strauss and Barney Glaser in 1967 [21]. Its core idea is that researchers do not have theoretical assumptions before the beginning research, but organize, summarize, and analyze the original data through standardized and systematic operations, finally establishing a theoretical model from the bottom up. The method has a clear process, strong operability, scientific and normative characteristics. Foley et al. [22] used GT to capture and analyze health care experiences. Andrews et al. [23] applied GT to explore nurses’ experience of self-care and self-compassion and how this may relate to compassionate care giving towards patients. A fuzzy analytical hierarchy process (FAHP) is a quantitative analysis method, which introduces the idea of fuzzy mathematics based on the analytical hierarchy process (AHP), and can effectively reduce interference from the subjectivity of decision makers’ judgments and preferences [24]. Entropy weight method (EWM) is a commonly used objective weighting method that the objective weight of each indicator is assigned according to the degree of variation of various indicators [25, 26]. Combining FAHP and EWM (FAHP-EWM), the subjective weight is determined by FAHP and the objective weight is determined by EWM, which can improve the scientificity and accuracy of the weight evaluation results. In this study, GT and FAHP-EWM was used to construct the framework for evaluating GOH-IDI with various indicators, to further improve One Health governance process.

## Methods

### The selection of indicators

The evaluation framework of GOH-IDI was constructed mainly using GT and expert interviews. With the GT method, data were collected from published literature and public database, and analyzed simultaneously. Twenty-eight experts working in the field of human medicine, animal medicine, environmental science, policy making and public administration, sociology, and psychology were interviewed. All of these international experts have a master degree or higher level of education, and most of them have a vice-senior or senior level of profession with more than 5 years of work experience in their respective fields (Table 1).

**Table 1:** Basic information of interviewed experts

Characteristics	Total (*n* = 28)	Proportion (%)
Gender		
Male	16	57.1
Female	12	42.9
Age		
21–35 years old	7	25.0
36–50 years old	13	46.4
≥ 51 years old	8	28.6
Education level		
Doctoral degree	22	78.6
Master degree	6	21.4
Professional level		
Senior level	14	50.0
Vice-senior level	11	39.3
Middle level	3	10.7
Geographical origin		
Asia	12	42.9
Europe	8	28.6
America	3	10.7
Africa	4	14.3
Oceania	1	3.6
Research area		
Human medicine	15	53.6
Veterinary science	10	35.7
Environmental science	9	32.1
Policy making	8	28.6
Public administration	9	32.1
Sociology	4	14.3
Psychology	3	10.7

**Fig. 1:**
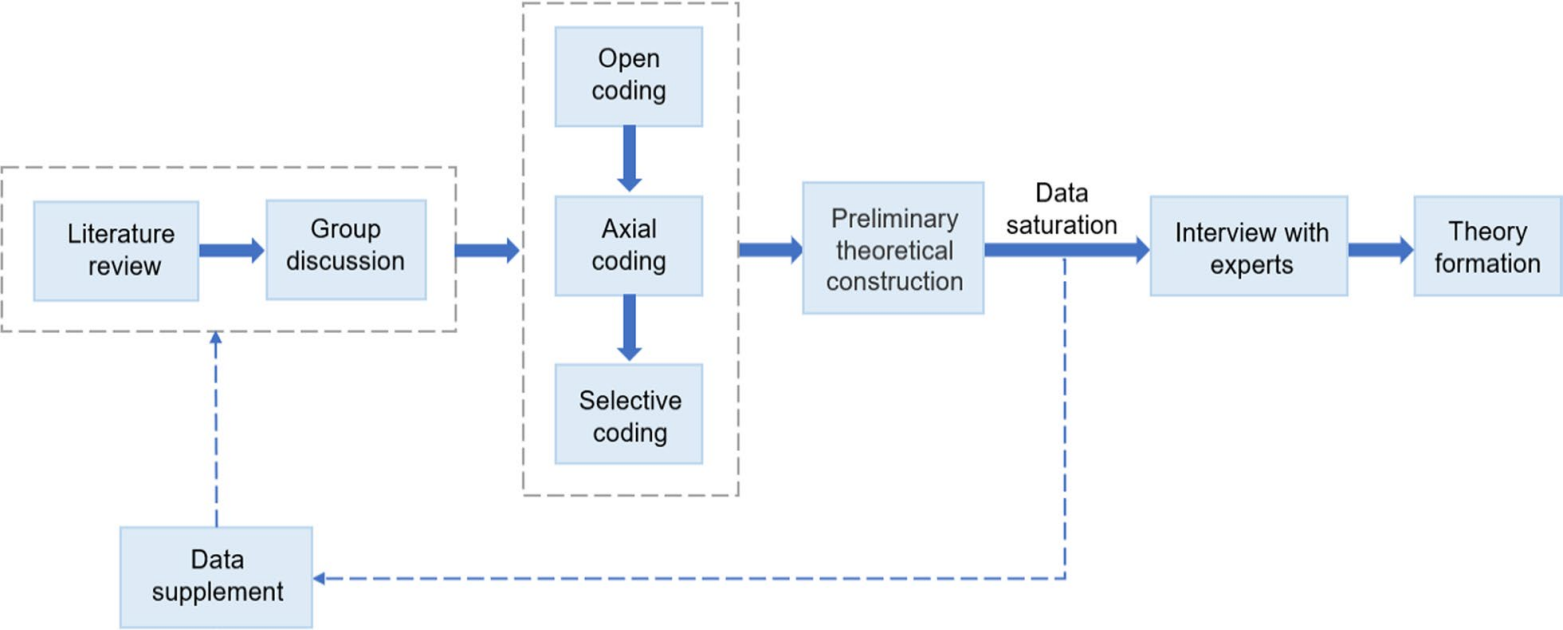
Process of indicators selection and framework establishment

The accuracy and reliability of the qualitative research phase depend on the research process. Therefore, two researchers coded and interviewed experts to assess the coding results and ensure the accuracy of the data. When no new concepts appeared after reading new literature, the research was deemed to have reached a state of theoretical saturation, which ensured the reliability of the data. Figure 1 shows the process of the qualitative research phase of this study.

#### The basis of GT

At the first stage of this study, the authors searched the PubMed and ISI Web of science databases using “One Health and indicator”, “One Health and index”, “One Health and metrics”, and “One Health and evaluation” as the keywords. The authors initially selected 103 articles related to the One Health assessment indicators by reviewing article abstracts and references to extract additional relevant articles. Subsequently, the authors consulted 12 global authoritative databases, such as the Sustainable Development Goal (SDG) database, the IHME-GBD, and the EPI. Then, the authors developed five indicator inclusion criteria for the GOH-IDI: First, indicators can accurately and truthfully measure or reflect the health status of a particular field. Second, the data used for indicators can be obtained from authoritative institutions. Third, the data used for indicators can cover most countries in the world. Fourth, the data used for indicators can be updated on time. Fifth, the data used for indicators should be measured in a uniform way to facilitate comparisons between countries. Based on the above inclusion criteria for the indicators, a total of 82 studies were included.

#### The process of GT

At the second stage of the study, GT was used to construct the evaluation indicator framework of GOH-IDI based on the 82 studies identified in the first stage. For the GT, data collection and analysis were carried out concurrently. Thus, data analysis began from reading the first document, and reading the second document was based on the analysis of the previous document. Subsequently, reading following documents was based on the analysis of all prior documents. No new concepts or categories were found in the data analysis after the 69th article. However, to ensure theoretical saturation, the researchers read the other 13 articles and analyzed the relevant data. The coding in the GT included three steps: open coding, axial coding, and selective coding [27]. Each step is described in more detail below.

##### Open coding

The first coding step in the GT was open coding. The authors identified and coded concepts (open codes, i.e., third-level indicators) related to human, animal and environmental health from the literature. Each document was analyzed and coded before moving to the next document. New literature was constantly compared to existing literature to identify new open codes (Table 2). Throughout the entire open coding process, we not only identified open codes in the data, but also tried to generate categories of extracted open codes, and open codes with similar meanings were divided into the same category. Such classification helped identify associations hidden among the open codes and guided us in coding the remaining studies.

**Table 2:** Examples of open codes extracted from the literature

Source	Open codes														
	Emerging rival diseases	Neonatal health	Cardiovascular disease	Tuberculosis	Neoplasms	Controlled solid waste	Overexploited or collapsed fish stocks	Air quality and climate change	Tree cover loss	Wastewater treatment	Trawling or dredging fish	Diseases of domestic animal	Cattle milk production efficiency	Health care system	Diseases of wild animal
SDG [28]		√	√	√	√			√		√					
IHME-GBD [29]		√	√	√	√										
EPI [30]						√	√	√	√	√	√				
OIE-WAHIS [31]												√			√
Our World in Data [32]													√		
Hanin et al. [33]	√			√											
Naylor et al. [34]								√							
Dutkiewicz et al. [35]		√	√		√					√					
Rüegg et al. [36]														√	
Alfonso Sanchez et al. [37]			√	√											

*SDG* Sustainable Development Goal, *IHME-GBD* Institute for Health Metrics and Evaluation the global burden of diseases, *EPI* environmental performance index, *OIE-WAHIS* OIE World Animal Health Information System

##### Axial coding

The second coding step was axial coding. In open coding, data were shredded to better identify the open codes. In axial coding, the open codes were reorganized in a new format by associating the categories and open codes with each other. Therefore, we associated categories with open codes and treated all interrelated categories as more abstract axial codes, i.e., second-level indicators. Therefore, following the axial coding, axial codes were identified to evaluate the main ideas of human, animal and environmental health.

##### Selective coding

The third coding step was selective coding. Selective coding is the process of linking core categories with open codes and axial codes, and forming the overall framework. In this step, all open codes and axial codes were integrated and organized around the core category (selective codes, i.e., first-level indicators) to form a complete evaluation indicator framework of GOH-IDI. The codes generated was reviewed, revised, and improved successively. If blank areas were found, theoretical sampling, i.e., literature review, was used to fill them.

#### Expert interviews

In the third stage, semi-structured interviews were conducted with 28 experts in human medicine, animal medicine, environmental science, policy making and public administration, sociology, and psychology, among other fields, based on the evaluation indicator framework of GOH-IDI initially formed by the GT in the second stage. We conducted two rounds of expert interviews. Experts were offered face-to-face interviews but, due to occasional limitations in public gatherings because of COVID-19 restrictions and personal preference, interviews via Zoom were also used. First, we described the concept and importance of the GOH-IDI to the experts, and introduced the indicator inclusion criteria, inclusion process and preliminary indicator framework to the experts in detail. Then, we invited experts to evaluate, guide and improve our indicator system. Each expert was interviewed lasted approximately 10 to 20 min (Additional file 1). The interviews were audio-recorded (with experts consent) and we also took notes during the interviews. We revised the indicator framework based on expert opinions to make it more scientific and reasonable. After two rounds of expert interviews, the final GOH-IDI evaluation indicator framework was determined.

### Setting of indicator weights

#### FAHP

In this section, we used a FAHP for quantitative assessments. The FAHP is a decision-making method based on the traditional AHP, which takes into account the fuzziness of people's judgment of complex things and introduces the fuzzy consistent matrix [24, 38]. We used a FAHP to determine the weight of the second-level indicators, and set the first-level and third-level indicators with equal weights. Based on the established evaluation indicator framework of GOH-IDI, a questionnaire was developed to assess the importance of indicators according to the comparisons of the relative importance of second-level indicators to first-level indicators. The questions involved in the questionnaire compared every two indicators of all second-level indicators (For example, there is such a question in the questionnaire: “Which do you think is more important in human health assessment, ‘Reproductive, Maternal, New-born, and Child Health’ or ‘Infectious Diseases’?”). The questionnaire for judging the importance of indicators was distributed to 42 experts in the fields of health-related, and we analyzed their opinions on the relative importance of indicators.

First, R 4.0.0 software (R Development Core Team; R foundation for Statistical Computing; Vienna, Austria) was used to generate a judgment matrix ${R}_{a\times a}^{b}$ according to the opinions of different experts, as shown in Eq. (1). *a* indicates that there are *a* indicators, and *b* indicates that there are *b* experts. ${r}_{ie}$ represents the importance of indicator *i,* relative to indicator *e*. Then, using Eq. (2) to calculate weight vector. Finally, normalizing the weight vector to calculate weights of the second-level indicators, as shown in Eq. (3).

1 $${{\varvec{R}}}_{a\times a}^{b}=\left[\begin{array}{cccc}{r}_{11}& {r}_{12}& \cdots & {r}_{1a}\\ {r}_{21}& {r}_{22}& \cdots & {r}_{2a}\\ \vdots & \vdots & ...& \vdots \\ {r}_{a1}& {r}_{a2}& \cdots & {r}_{aa}\end{array}\right]=({r}_{ie})$$

2 $${{\varvec{\omega}}}_{i}=\frac{{\left(\prod_{j=1}^{a}{r}_{ie}\right)}^\frac{1}{a}}{{\sum }_{k=1}^{a}{\left(\prod_{j=1}^{a}{r}_{ke}\right)}^\frac{1}{a}}$$

3 $${{\varvec{W}}}_{i}=\frac{{{\varvec{\omega}}}_{i}}{{\sum }_{e=1}^{a}{{\varvec{\omega}}}_{e}}$$

#### EWM

The entropy weight method uses the entropy value to calculate the variation degree of the indicator, and assigns weights according to the variation degree of each indicator. When the value difference of the evaluation object on a certain indicator is large, the entropy value is small, indicating that the information provided by the indicator is large, and the weight of the indicator should be large, that is, the judgment matrix composed of the evaluation indicator value determines the weight of each evaluation indicator [39, 40]. The specific steps are as follows:

Step1: Data standardization, as shown in Eq. (4).

4 $${{\varvec{r}}}_{ij}=\frac{{{\varvec{x}}}_{ij}-{{\varvec{x}}}_{jmin}}{{{\varvec{x}}}_{jmax}-{{\varvec{x}}}_{jmin}}, \left(i=\mathrm{1,2},\dots ,m;j=\mathrm{1,2},\dots ,n\right),$$

Step2: If there are *m* objects to be evaluated and *n* indicators to be evaluated, the dimensionless data matrix ${\varvec{R}}$ is constructed according to Eq. (4), as shown in Eq. (5).

5 $${\varvec{R}}=\left[\begin{array}{cccc}{r}_{11}& {r}_{12}& \cdots & {r}_{1n}\\ {r}_{21}& {r}_{22}& \cdots & {r}_{2n}\\ \vdots & \vdots & ...& \vdots \\ {r}_{m1}& {r}_{m2}& \cdots & {r}_{mn}\end{array}\right]$$

Step3: Calculate the proportion of the *i*-th object under the *j*-th indicator, as shown in Eq. (6).

6 $${{\varvec{P}}}_{ij}=\frac{{{\varvec{r}}}_{ij}}{{\sum }_{i=1}^{m}{{\varvec{r}}}_{ij}}$$

Step4: Solve the entropy value under the *j*-th indicator, as shown in Eq. (7).

7 $${{\varvec{e}}}_{j}=-\frac{1}{\mathrm{ln}m}{\sum }_{i=1}^{m}{{\varvec{P}}}_{ij}\mathrm{ln}{{\varvec{P}}}_{ij}$$

Step5: Calculate the entropy weight coefficient of the *j*-th indicator, as shown in Eq. (8).

8 $${{\varvec{W}}}_{j}=\frac{1-{{\varvec{e}}}_{j}}{{\sum }_{i=1}^{n}(1-{{\varvec{e}}}_{j})}$$

#### FAHP-EWM

The integrated weight of the indicator is determined by minimizing the sum of squared deviations. It is assumed that the integrated weight is ${{\varvec{W}}}_{ij}$, the subjective preference coefficient is *β*, the weight calculated by FAHP is ${{\varvec{W}}}_{i}$, and the weight calculated by EWM is ${{\varvec{W}}}_{j}$. The formula for calculating the integrated weight as shown in Eq. (9). The formula of objective function is established by minimizing the sum of squared deviations, as shown in Eq. (10). Substituting Eq. (9) into Eq. (10) gives us *β* = 0.5. Therefore, the formula for calculating the integrated weight can be regarded as Eq. (11).

9 $${{\varvec{W}}}_{ij}=\beta {{\varvec{W}}}_{i}+(1-\beta ){{\varvec{W}}}_{j}$$

10 $$min{\varvec{Z}}={\sum }_{i=1}^{n}\left[\left({{\varvec{W}}}_{ij}-{{\varvec{W}}}_{i}\right)+({{\varvec{W}}}_{ij}-{{\varvec{W}}}_{j})\right]$$

11 $${{\varvec{W}}}_{ij}=0.5{{\varvec{W}}}_{i}+0.5{{\varvec{W}}}_{j}$$

## Results

### Revision of GOH-IDI evaluation indicators

Based on the GT, we used a literature review and group discussions to preliminarily develop a three-level evaluation indicator framework of GOH-IDI, which consisted of three selective codes, 19 axial codes, and 79 open codes. Subsequently, through expert interviews, the indicators were further integrated and streamlined according to the inclusion criteria. In open codes, we added one code and deleted 19 codes according to experts’ suggestion. We added “COVID-19”, and deleted “Hepatitis B virus”, “Physical sexual or physiological violence”, “Prevalence of emerging infectious disease”, “Severity of emerging infectious disease”, “Emergency response capacity”, “Companion animals”, “Animals used for draught and recreation”, “Animal sentience”, “Animal protection laws”, “Laws apply to animals used in farming”, “Laws apply to animals in captivity”, “Laws apply to animals used in scientific research”, “Laws that apply to wild animals”, “Discarded Fish”, “Threatened Bird Species”, “Threatened Mammal Species”, “Threatened Fish Species”, “Threatened Plant Species” and “Marine Protected Areas”. In axial codes, we integrated “Air Quality” and “Climate Change” into “Air Quality and Climate Change” in response to experts’ suggestion. We changed “Ecosystem Services” to “Land Resources”, “Waste management” to “Hazardous Chemicals”, and “Animal feeding” to “Animal Nutritional Status”, and deleted “Public Health Emergency of International: emerging infectious disease”, “Marine life”, and “Fisheries”.

### Final evaluation indicator framework of GOH-IDI

The final evaluation indicator framework of GOH-IDI was constructed using three selective codes, 15 axial codes, and 61 open codes. The 61 concepts, including “Maternal Health”, “Neonatal Health”, “Child Health”, and “Adolescent Fertility” were based on open codes (third-level indicators) extracted from the literature and group discussions. The 15 categories, including “Reproductive, Maternal, New-born and Child Health”, “Infectious Diseases”, “Non-communicable Diseases”, and “Mental Health” were based on axial codes (second-level indicators) extracted from the open codes. “Human Health”, “Animal Health”, and “Environmental Health” were the core categories, i.e., selective codes (first-level indicators) of the evaluation indicator framework of GOH-IDI, based on axial code induction (Table 3).

**Table 3:** Evaluation indicator framework of GOH-IDI based on GT and FAHP-EWM

Selective codes (First-level indicators)	Weight (%)	Axial codes (Second-level indicators)	FAHP weight (%) ${W}_{i}$	Entropy value ${e}_{j}$	EWM weight (%) ${W}_{j}$	Integrated weight (%) ${W}_{ij}$	Open codes (Third-level indicators)	Weight (%)
1. Human Health	33.33	1.1 Reproductive, Maternal, New-born, and Child Health	20.63	1.47	17.37	19.00	1.1.1 Maternal Health	25.00
							1.1.2 Neonatal Health	25.00
							1.1.3 Child Health	25.00
							1.1.4 Adolescent Fertility	25.00
		1.2 Infectious Diseases	19.53	1.54	19.99	19.76	1.2.1 Tuberculosis	20.00
							1.2.2 HIV	20.00
							1.2.3 Malaria	20.00
							1.2.4 Neglected Tropical Diseases	20.00
							1.2.5 COVID-19	20.00
		1.3 Non-communicable Diseases and Mental Health	15.88	1.49	18.16	17.02	1.3.1 Cardiovascular Disease	20.00
							1.3.2 Neoplasms	20.00
							1.3.3 Diabetes Mellitus	20.00
							1.3.4 Chronic Respiratory Disease	20.00
							1.3.5 Suicide	20.00
		1.4 Injuries and Violence	13.49	1.27	9.95	11.72	1.4.1 Road Traffic	33.33
							1.4.2 Unintentional Poisoning	33.33
							1.4.3 Homicide	33.33
		1.5 Universal Health Coverage and Health Systems	17.47	1.51	19.01	18.24	1.5.1 Health Coverage	25.00
							1.5.2 Research and Development Expenditures on Health Issues	25.00
							1.5.3 Domestic Health Expenditures	25.00
							1.5.4 Infant Vaccination	25.00
		1.6 Health Risk	13.01	1.42	15.52	14.27	1.6.1 Unsafe or Unimproved Water, Sanitation and Hygiene	33.33
							1.6.2 Household Air Pollution	33.33
							1.6.3 Occupational Risks	33.33
2. Animal Health	33.33	2.1 Animal Epidemic Disease	31.87	2.01	46.69	39.28	2.1.1 Diseases of Domestic Animals	50.00
							2.1.2 Diseases of Wild Animals	50.00
		2.2 Animal Welfare, Relevant Regulations, and Policy Support	24.66	1.13	5.77	15.22	2.2.1 Overexploited or Collapsed Stocks Fish	50.00
							2.2.2 Trawling or Dredging Fish	50.00
		2.3 Animal Nutritional Status	17.36	1.13	5.81	11.59	2.3.1 Chicken Meat Production Efficiency	25.00
							2.3.2 Pig Meat Production Efficiency	25.00
							2.3.3 Cattle Production Efficiency	25.00
							2.3.4 Cattle Milk Production Efficiency	25.00
		2.4 Animal Biodiversity	26.11	1.90	41.72	33.91	2.4.1 Endemic Mammal Species	16.67
							2.4.2 Endemic Bird Species	16.67
							2.4.3 Endemic Amphibian Species	16.67
							2.4.4 Endemic Reef-forming Coral Species	16.67
							2.4.5 Endemic Freshwater Crab Species	16.67
							2.4.6 Endemic Shark and Ray Species	16.67
3. Environmental Health	33.33	3.1 Air Quality and Climate Change	23.82	1.66	21.44	22.63	3.1.1 Ambient Particulate Matter Pollution	20.00
							3.1.2 Household Solid Fuels	20.00
							3.1.3 Ambient Ozone Pollution	20.00
							3.1.4 Climate Risk	20.00
							3.1.5 Greenhouse Gas	20.00
		3.2 Land Resources	19.55	1.58	18.95	19.25	3.2.1 Area at Risk Elevation	20.00
							3.2.2 Tree Cover Loss	20.00
							3.2.3 Grassland Loss	20.00
							3.2.4 Wetland Loss	20.00
							3.2.5 Mineral Depletion	20.00
		3.3 Sanitation and Water Resources	20.68	1.68	22.24	21.46	3.3.1 Freshwater	33.33
							3.3.2 Clean Drinking Water	33.33
							3.3.3 Renewable Internal Freshwater Resources	33.33
		3.4 Hazardous Chemicals	17.52	1.56	18.12	17.82	3.4.1 Fertilizer Consumption	14.28
							3.4.2 Controlled Solid Waste	14.28
							3.4.3 SO_2_ Growth	14.28
							3.4.4 NO_X_ Growth	14.28
							3.4.5 Wastewater Treatment	14.28
							3.4.6 Electronic Waste	14.28
							3.4.7 Non-recycled Municipal Solid Waste	14.28
		3.5 Environmental Biodiversity	18.42	1.59	19.25	18.83	3.5.1 Protected Areas Representativeness	33.33
							3.5.2 Species Habitat	33.33
							3.5.3 Biodiversity Habitat	33.33

*GOH-IDI* global One Health Intrinsic Drivers index; *GT* Grounded Theory; *EWM* entropy weight method; *FAHP* fuzzy analytical hierarchy process

There were six axial codes for “Human Health”, which were “Reproductive, Maternal, New-born, and Child Health”, “Infectious Diseases”, “Non-communicable Diseases and Mental Health”, “Injuries and Violence”, “Universal Health Coverage and Health Systems”, and “Health Risk”. Human Health focused on health throughout the complete life cycle of the entire population, the health risks brought by animals and the external environment, and the role of the health system in ensuring Human Health.

There were four axial codes for “Animal Health”, which were “Animal Epidemic Disease”, “Animal Welfare, Relevant Regulations, and Policy Support”, “Animal Nutritional Status”, and “Animal Biodiversity”. Animal Epidemic Disease affected Animal Nutritional Status, and Animal Nutritional Status reacted to Animal Epidemic Disease. Animal Biodiversity was affected by Animal Welfare, but was more related to macro ecological environments.

There were five axial codes for “Environmental Health”, which were “Air Quality and Climate Change”, “Land Resources”, “Sanitation and Water Resources”, “Hazardous Chemicals”, and “Environmental Biodiversity”. The discharge of hazardous chemicals had an impact on all aspects of Environmental Health. Climate change, loss of land resources, ecological construction and water resources are concrete manifestations of Environmental Health.

### Indicator weights

In this study, the first-level and third-level indicators were set with equal weights, and the weight of second-level indicators were determined by FAHP-EWM. Thus, the weights of the first-level indicators of the evaluation indicator framework of GOH-IDI, “Human Health”, “Animal Health”, and “Environmental Health” were 33.33%. Among the second-level indicators of “Human Health”, “Infectious Diseases” had the highest weight (19.76%), while the weights of the other indicators from highest to lowest were “Reproductive, Maternal, New-born, and Child Health” (19.00%), “Universal Health Coverage and Health Systems” (18.24%), “Non-communicable Diseases and Mental Health” (17.02%), “Health Risk” (14.27%), and “Injuries and Violence” (11.72%). Among the second-level indicators of “Animal Health”, “Animal Epidemic Disease” (39.28%) and “Animal Biodiversity” (33.91%) had higher weights, while “Animal Welfare, Relevant Regulations, and Policy Support” (15.22%) and “Animal Nutritional Status” (11.59%) had lower weights. Among the second-level indicators of “Environmental Health”, “Air Quality and Climate Change” had the highest weight (22.63%), while the weights of the other indicators from highest to lowest were “Sanitation and Water Resources” (21.46%), “Land Resources” (19.25%), “Environmental Biodiversity” (18.83%), and “Hazardous Chemicals” (17.82%).

### Indicator pathways

**Fig. 2:**
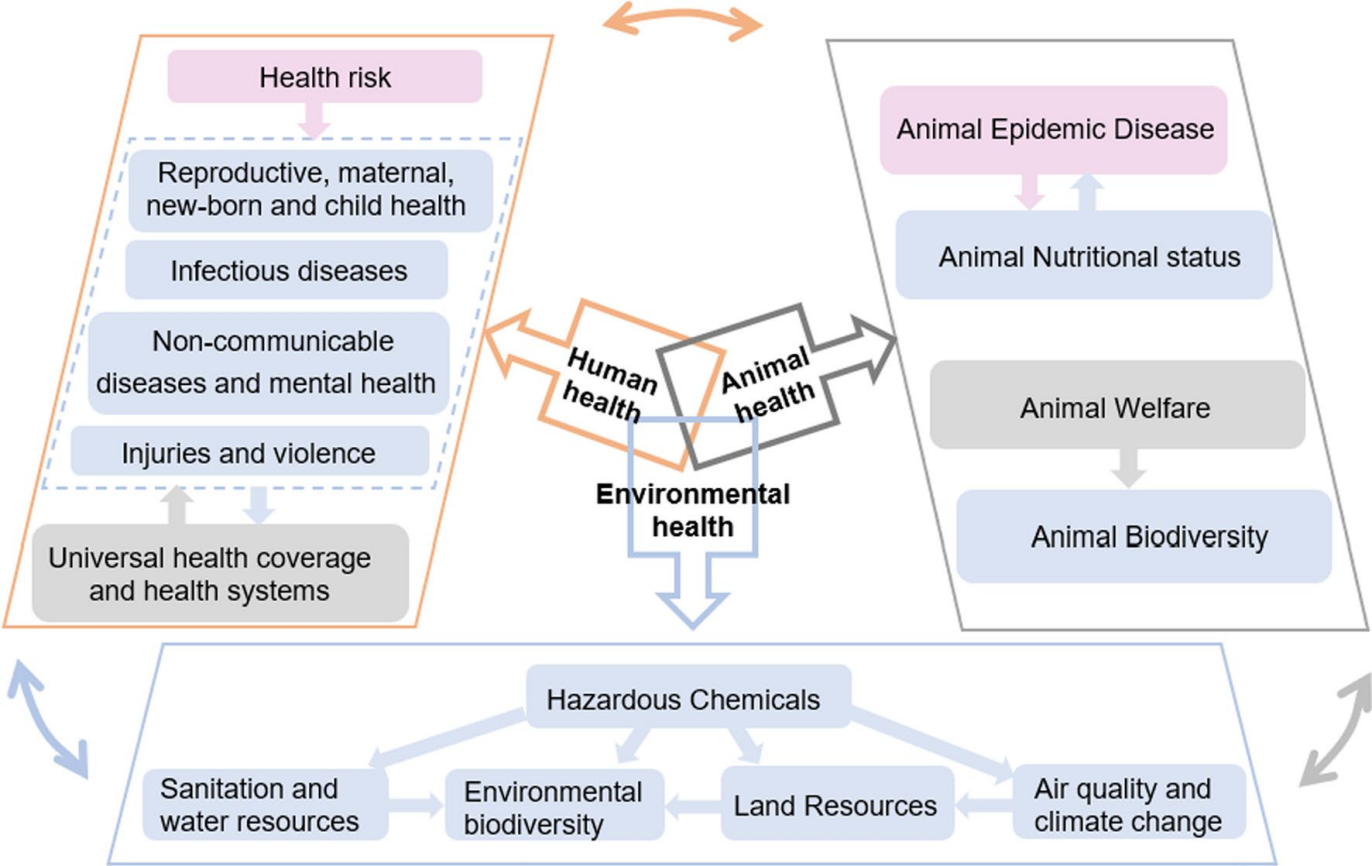
Relationships for the evaluation indicators of GOH-IDI. According to the “structure-process-result” model, the second-level indicators of the indicator framework of GOH-IDI were divided into different categories: gray represents structural indicators, pink represents process indicators, and blue represents outcome indicators

According to the concept of One Health, human health, animal health and environmental health restricted and promoted each other to form an organic and unified One Health system (Fig. 2). According to the “structure-process-result” model, the constructed One Health indicators were divided into structural indicators, process indicators, and outcome indicators. “Structure” refers to infrastructure, “process” refers to intervention measures, and “outcome” refers to post-intervention performance.

In Human Health, the four second-level indicators, “Reproductive, Maternal, New-born, and Child Health”, “Infectious Diseases”, “Non-communicable Diseases and Mental Health”, and “Injuries and Violence”, were outcome indicators, which reflected the whole population life cycle health, and were the core indicators of Human Health. “Universal Health Coverage and Health Systems” was a structural indicator, which reflected the guarantee of health system on human health. “Health Risk” was a process indicator that was composed of indicators of the impact of the external environment on human health. “Health Risk” can affect the core indicators, and “Universal Health Coverage and Health Systems” was affected by the core indicators, and can react to the core indicators.

In Animal Health, “Animal Epidemic Disease” was a process indicator, and the most important part of animal health. “Animal Welfare, Relevant Regulations, and Policy Support” were structural indicators reflecting policies and regulations related to animal welfare. “Animal Nutrition Status” and “Animal Biodiversity” were outcome indicators that directly reflected animal health. Animal nutrition status and animal epidemic disease affected each other. A decline in animal nutrition status can lead to a decline in animal immunity and an increase in the number of reported animal epidemic diseases. Animal epidemic disease could also affect the intake of nutrients in food. Although “Animal Biodiversity” was affected by animal welfare, it is more related to the macro ecological environment.

In Environmental Health, “Air Quality and Climate Change”, “Land Resources”, “Sanitation and Water Resources”, “Hazardous Chemicals”, and “Environmental Biodiversity” were all outcome indicators that directly reflected the health of the environment. The discharge of hazardous chemicals can affect all steps and should be controlled emphatically. Air quality and climate change, loss of land resources, ecological environment construction and water resources, and environmental biodiversity were all concrete manifestations of environmental health. Damage to air quality could lead to extreme weather, which in turn affected land resources. The destruction of land resources will lead to habitat loss for animals and plants, and affect environmental biodiversity. Ultimately, all environmental pressures will be transferred to animal and human health.

## Discussion

Previous studies have proposed GOHI, which provides an evaluation method for One Health governance process in various countries around the world, and illustrates the determinants and contributing factors of the achievements of One Health governance. The intrinsic drivers index, which emphasize the synergistic development of human, animal and environmental health, are an important part of GOHI. In-depth analysis of intrinsic drivers index can highlight the governance elements and key issues of human, animal and environmental health, and help to optimize the allocation of health resources and promote the process of sustainable development.

GT is a bottom-up inductive research method that aims to establish theories based on empirical data. GT is performed by iterating data collection and analysis by performing continuous comparisons, extracting concepts reflecting research results in the process of continuous comparison, developing categories and the correlations between categories, and combining all such data into theories [41]. Theoretical sampling continues until the data are saturated and the theory is complete. The results from the method were not only based on practical experience, but also better than practice. When the results are applied in practice, its advantages will be more prominent. Expert interviews can qualitatively determine the scientificity and rationality of the index system. Through our elaboration of the construction process of the indicator system, experts replaced, integrated, added and deleted some indicators according to their practical experience in their respective fields, the representativeness and completeness of indicators, etc., until all the interviewed experts considered the indicator system saturated (Additional file 2). A FAHP can avoid the influence of human subjectivity and objectively determine the weights of indicators. It introduces the idea of fuzzy mathematics based on the AHP, which can divide the complicated factors of the evaluated object into an orderly hierarchical structure according to the interaction, influence, and subordinate relationship to obtain the weights of the indicators. It can also effectively reduce interference from the subjectivity of decision makers’ judgments, preferences, and understanding of the problem being solved [42]. EWM is a typical objective weight method, only complete data samples are required, and the sample size is not high, but the accuracy is high. Combining subjective method with objective method to determine indicators weight has been widely used in various fields. For example, Xi et al. [43] evaluated the capability of municipal solid waste separation in China based on AHP-EWM. Song et al. [44] evaluated the fraud vulnerability of Wuchang rice industrial chain in China based on AHP-EWM. Therefore, to make the evaluation indicator framework of GOH-IDI not only reflect the connotation of One Health and minimize the one-sidedness of indicator weights due to human subjectivity, the GT, expert interviews and FAHP-EWM was used to establish the evaluation indicator framework and render it more scientific and objective.

Based on the GT and FAHP-EWM, this study constructed a three-level evaluation indicator framework of GOH-IDI, which was composed of three selective codes, 15 axial codes, and 61 open codes using literature searches, group discussions, and expert interviews. There were six second-level indicators and 24 third-level indicators in Human Health, among which, “Infectious Diseases” had the highest weight (19.76%). There were four second-level indicators and 14 third-level indicators in Animal Health, among which, “Animal Epidemic Disease” had the highest weight (39.28%). There were five second-level indicators and 23 third-level indicators in Environmental Health, among which, “Air Quality and Climate Change” had the highest weight (22.63%). Existing health-related evaluation systems around the world, such as the Sustainable Development Report released by the United Nations, summarize the current development trends of 17 Sustainable Development Goals in each country from three aspects: economic development, social progress, and environmental protection [45]. The Global Health Security Index (GHS Index), jointly developed by the Nuclear Threat Initiative (NTI) and the Johns Hopkins Center for Health Security, focuses on the ability of countries to prevent and control pandemics from six aspects: prevention, detection and reporting, rapid response, health systems, compliance with international norms, and risk environment [46]. It can be seen that these existing health-related evaluation systems only focus on specific scientific fields of One Health, and thus, the research on One Health is fragmented and limited. This study explored the evaluation indicator framework of GOH-IDI from the aspects of human health, animal health and environmental health, most of the selected indicators are outcome indicators, which can directly reflect the practice process of human, animal and environmental health, and the indicators are universal, balanced and scientific. This study reported the establishment process for indicator framework of GOH-IDI, and established a tool for scientifically measuring the development level of human, animal and environmental health in different regions to evaluate the progress and development of One Health capacity building throughout the world.

Through literature review and expert interviews, Hanin et al. [33] showed that collaborative development between multiple disciplines is crucial in One Health governance, and its weight accounts for 70% of One Health governance. Hanin et al. proposed that currently, collaboration between human health and animal health teams is gradually increasing, but collaboration with other disciplines is not enough. Therefore, different countries, disciplines and institutions should be combined to enhance the capacity of One Health building in an integrated manner. Bordier et al. [47] constructed an evaluation matrix to measure the cross-sector collaboration of One Health in order to evaluate the quality of One Health cross-sector collaboration. It was found in this study that the literature related to human health indicators was the most abundant followed by that related to environmental health and the literature related to animal health indicators were least abundant, which was consistent with the research results of Vreeland et al. [48] Moreover, Vreeland et al. also proposed that in health-related journals, only 6.8% of journals included articles related to human, animal and environmental health at the same time. Therefore, we gave equal weights to Human Health, Animal Health, and Environmental Health in the GOH-IDI (i.e., 33% each), reflecting the importance of multidisciplinary collaborative development in the concept of One Health.

Previous studies used FAHP to determine the weight of second-level indicators of GOH-IDI. This study used FAHP-EWM to verify and optimize the weight of second-level indicators of GOH-IDI [16]. In human health, compared with FAHP, FAHP-EWM improved the weight of “Infectious Diseases”, “Non-communicable Diseases and Mental Health”, “Universal Health Coverage and Health Systems”, and “Health Risk”. Numerous studies have shown that non-communicable diseases kill more than 41 million people every year, accounting for 71% of all deaths globally [49–51], and with economic development, there is an increasing focus on chronic diseases, mental illnesses, and Universal Health Coverage [52–54]. In animal health, compared with FAHP, FAHP-EWM improved the weight of “Animal Epidemic Disease” and “Animal Biodiversity”. Many studies indicated that animal epidemic disease pose a great threat to animal health and the development of breeding industry and should be paid attention to by the public [55, 56]. The issue of animal biodiversity is also receiving increasing attention. Studies have shown that the most unique ecological functions in nature are mostly derived from rare animals, and the protection of animal biodiversity is crucial to the resilience and survival of ecosystems, and these rare animals may play an important role for One Health in the future [57–59]. In environmental health, the indicator weight calculated by FAHP-EWM is basically consistent with that of FAHP, so it can be considered that the GOH-IDI second-level indicator weights obtained by these studies are scientific.

There were some deficiencies in this study. Firstly, after determining the indicator framework through grounded theory and experts interview method, the Delphi method can also be used to improve the indicator framework. Secondly, this study only describes the construction of the indicator system, the intrinsic dynamics of GOH-IDI can be further studied in the future. Thirdly, the evaluation indicator framework of GOH-IDI proposed in this study was based on preliminary research and exploration, which needs to be further improved by combining evaluation indicator framework of GOH-IDI with the progress of One Health governance in each country. Fourthly, due to the poor compatibility of the research team with multiple languages, the studies retrieved for this indicator framework were only in English, which did not allow for a comprehensive assessment of studies in other languages, such as French, German, and Russian, among others.

## Conclusions

Based on the GT and FAHP-EWM, this study constructed a three-level evaluation indicator framework of GOH-IDI, which was composed of three first-level indicators, 15 second-level indicators, and 61 third-level indicators using literature searches, group discussions, and expert interviews. Most of the selected indicators in evaluation framework are outcome indicators, which can directly reflect the practice process of human, animal and environmental health, and can provide scientific reference for scientific measurement of human, animal and environmental health development in various regions. The evaluation framework of GOH-IDI provides an overall framework for “what to implement” and “how to improve” in the One Health governance, and can also be used as a guide for planning the governance of human, animal and environmental health throughout the world.

## Supplementary Information

**Additional file 1.** Expert interviews guide.

**Additional file 2.** Selected quotations and corresponding modification.

## Data Availability

The data used and/or analyzed during the current study are available from the corresponding author on reasonable request.

## Abbreviations

GOHI: Global One Health index
GOH-IDI: Global One Health Intrinsic Drivers index
WHO: World Health Organization
OIE: World Organization for Animal Health
FAO: Food and Agriculture Organization of the United Nations
UNEP: United Nations Environment Programme
OHHLEP: One Health High Level Expert Panel
SDR: Sustainable development report
GBD: Global burden of diseases
EPI: Environmental performance index
OIE-WAHIS: OIE World Animal Health Information System
GT: Grounded theory
SDG: Sustainable Development Goal
WHO-GHO: World Health Organization-Global Health Observatory
IHME-GBD: Institute for Health Metrics and Evaluation the global burden of diseases
FAHP: Fuzzy analytical hierarchy process
AHP: Analytical hierarchy process
EWM: Entropy weight method
GHS Index: Global Health Security Index
NTI: Nuclear Threat Initiative
