# Supplementary material for: Establishment of an indicator framework for global One Health Intrinsic Drivers index based on the grounded theory and fuzzy analytical hierarchy-entropy weight method
Source: Infect Dis Poverty. 2022 Dec 8;11:121. doi: 10.1186/s40249-022-01042-3 (PMC9733012; doi:10.1186/s40249-022-01042-3)
Supplement: Supplementary file 1 — Additional file 1. Expert interviews guide. [file 40249_2022_1042_MOESM1_ESM.docx]

**Additional file 1**

**Expert Interviews Guide**

1. Welcome (5 minutes)

2. Research background (5 minutes)

3. Research purpose (5 minutes)

4. Expert interviews purpose (5 minutes)

5. Description of establishment of the indicator framework for GOH-IDI (10–15 minutes)

6. Ask the experts for their opinion (10–20 minutes)

Q1. Do you think the indicators we selected meet the indicator inclusion criteria of GOH-IDI? Please talk about your suggestion.

Q2. Based on your practical experience, what indicators do you think should be added or deleted in order to better evaluate human health? Please talk about your suggestion.

Q3. Based on your practical experience, what indicators do you think should be added or deleted in order to better evaluate animal health? Please talk about your suggestion.

Q4. Based on your practical experience, what indicators do you think should be added or deleted in order to better evaluate environmental health? Please talk about your suggestion.

7. Thank you
