# Supplementary material for: Establishment of an indicator framework for global One Health Intrinsic Drivers index based on the grounded theory and fuzzy analytical hierarchy-entropy weight method
Source: Infect Dis Poverty. 2022 Dec 8;11:121. doi: 10.1186/s40249-022-01042-3 (PMC9733012; doi:10.1186/s40249-022-01042-3)
Supplement: Supplementary file 2 — Additional file 2. Selected quotations and corresponding modification. [file 40249_2022_1042_MOESM2_ESM.docx]

**Additional file 2**

**Selected quotations and corresponding modifications**

| **Selected quotations** | **Corresponding modifications** |
| --- | --- |
| One Health should pay more attention to results-oriented indicators. | Change “Animal feeding” to “Animal Nutritional Status”. |
| There are a lot of indicators, the weight is difficult to set, can be streamlined according to the indicator inclusion criteria. | Delete “Marine life”. |
| COVID-19 can also be used to evaluate total health, and most countries have collated data on COVID-19, which can be considered as an indicator to be included in the index system. This indicator is very representative. | Add “COVID-19”. |
| It is recommended to check the completeness of the indicator data. | Delete “Hepatitis B virus” and “Physical sexual or physiological violence”. |
| There are many independent elements in the index system, so we should pay attention to the connection and logic between the indicators. | Integrated “Air Quality” and “Climate Change” into “Air Quality and Climate Change”. |
| There are many indicators of meaning repetition. It is suggested that the indicators of meaning repetition be merged or deleted. | Delete “Public Health Emergency of International: emerging infectious disease”. |
| If COVID-19 is included, "Public Health Emergency of International Concern: Emerging infectious diseases" can be deleted to streamline the indicators. | Delete “Prevalence of emerging infectious disease”, “Severity of emerging infectious disease” and “Emergency response capacity”. |
| Index selection is relatively scattered, lack of comprehensive, it is suggested to make the index more concentrated. “Fisheries” can be replaced by animal welfare. | Delete “Fisheries”. |
| The discharge of hazardous chemicals will affect many aspects of environmental health and should be paid more attention to. | Change “Waste management” to “Hazardous Chemicals”. |
| In environmental health, it is suggested to add some indicators related to sustainable development of land resources, such as tree cover loss, grassland loss and wetland loss, etc., which can be summarized into the category of land resources. | Change “Ecosystem Services” to “Land Resources”. |
| Indicators such as the Percentage of fish caught from overexploited or collapsed stocks are recommended. There are many indicators related to animal welfare. It is suggested to simplify the indicators evaluating similar areas. | Delete “Animal sentience”, “Animal protection laws”, “Laws apply to animals in captivity”, “Laws apply to animals used in scientific research”, “Laws that apply to wild animals”, and “Discarded Fish”. |
| Animal health is more concerned with whether indicators have underlying data. | Delete “Companion animals”, “Animals used for draught and recreation”, and “Laws apply to animals used in farming”. |
| There are a lot of repetitive meaning indicators in animal biodiversity. For example, a representative indicator can be used to describe the threat of mammals. | Delete “Threatened Bird Species”, “Threatened Mammal Species”, and “Threatened Fish Species”. |
| It is suggested to delete some indicators of environmental health and consider the part of biodiversity. | Delete “Threatened Plant Species”, and “Marine Protected Areas”. |
| Some indicators are not elements, but the relationship between two indicators, which needs to be noted. | Delete “Animal feeding”. |
| There are too many third-level indicators. It is recommended to delete the third-level indicators. | Make the third-level indicators more representative. |
| "Public Health Emergency of International Concern: Emerging infectious diseases" can be incorporated into other second-level indicators. | Delete "Public Health Emergency of International Concern: Emerging infectious diseases". |
| “Prevalence of emerging infectious disease”, “Severity of emerging infectious disease”, and “Emergency response capacity” can be represented by a comprehensive indicator. | Delete “Prevalence of emerging infectious disease”, “Severity of emerging infectious disease” and “Emergency response capacity”. |
